# Modifying Tobacco and Cannabis Waste Perceptions and Behavior Among Young Adults: Protocol for a Randomized Controlled Trial

**DOI:** 10.2196/79525

**Published:** 2025-12-04

**Authors:** Kim Pulvers, AB Nordskog, Hadley Shearer, Cheyenne Smith, Susan L Stewart, Thomas E Novotny, Elisa K Tong

**Affiliations:** 1 California State University, San Marcos San Marcos, CA United States; 2 University of California, Davis Davis, CA United States; 3 San Diego State University San Diego, CA United States

**Keywords:** cigarette butt, e-waste, vape, regulatory attitudes, plastic, corporate responsibility, environment, sustainability, smoking cessation, vaping cessation

## Abstract

**Background:**

Filtered cigarettes and vaped nicotine and cannabis negatively affect health and create nonbiodegradable, toxic waste from tobacco, e-cigarette, and cannabis waste (TECW). Creating awareness and action to address this public health issue requires expanded knowledge and understanding of TECW harms and more engagement with regulatory policies to reduce tobacco and cannabis use. This is the first study testing an intervention to modify TECW knowledge, perceptions, and behavior, including use of an innovative digital TECW tracking tool.

**Objective:**

This study tests the efficacy of an intervention to modify TECW knowledge, harm perceptions, attitudes, and behaviors regarding smoke- and vape-free university campus policies among young adults. We aim to develop an evidence-based educational intervention for use in community programs to address TECW.

**Methods:**

A 6-week randomized controlled trial was conducted at 2 sites representing the 2 public university systems in California from March 2023 to June 2025 with 406 students (aged 18-25 years) and compared brief tobacco, e-cigarette, and cannabis waste education (TECW Ed) plus tobacco, e-cigarette, and cannabis waste education plus motivational and behavioral support (TECW Ed+) with brief education only about TECW (TECW Ed). Participants were randomized 1:1 to each treatment group, stratified by site and tobacco or cannabis use status. Outcome measures include changes in knowledge, harm perceptions, regulatory attitudes, and behaviors regarding TECW, and engagement with smoke- and vape-free policies. Planned statistical analyses include models to assess knowledge, perceptions, and attitudes at weeks 2, 6, and 26 versus group, time, and a group × time interaction, controlling for site, tobacco or cannabis use status, demographics, and baseline level of outcomes. In addition, TECW Ed+ versus TECW Ed group comparison on mean number of Tracker reports between baseline and 6 weeks, and group comparison on mean engagement score at 6 weeks, will be conducted.

**Results:**

Data were collected from March 2023 to June 2025. Data analysis is expected to begin in late 2025, with final results anticipated for publication in summer 2026. Results will determine if brief educational videos accompanied by enhanced motivational and behavioral support increase knowledge of TECW’s environmental impact and change perceptions about cigarettes and vape products. We will determine whether such enhanced education increases engagement in smoke- and vape-free regulatory acceptance and improves outcomes of regulatory policies. This trial will be the first to test an intervention to modify tobacco and cannabis waste perceptions and behavior.

**Conclusions:**

This trial aims to determine whether additional motivational and behavioral support will change young-adult college students’ current knowledge of TECW and whether such support will motivate them to engage with regulatory policies to reduce TECW. If successful, scaling up this intervention may mobilize a large population of young adults to understand and advocate for policies that protect individual and environmental health.

**Trial Registration:**

ClinicalTrials.gov NCT05751369; https://clinicaltrials.gov/study/NCT05751369

**International Registered Report Identifier (IRRID):**

DERR1-10.2196/79525

## Introduction

### Background and Rationale

The global toxic, nonbiodegradable environmental waste from tobacco, e-cigarette, and cannabis waste (TECW) products is a pressing public health issue [1-4]. Upstream policy approaches [5] to address this increasingly recognized waste problem [6-11], such as banning sales of filtered cigarettes [12] and naming them as single-use plastics that may be addressed in a proposed international plastic treaty [13], are important for both environmental and human health reasons [14]. Awareness of the environmental impacts of e-cigarette [1,2,15-19] and cannabis vape products is also growing [2,19-22]. According to the Environmental Protection Agency (EPA), vape products cannot be safely discarded in household trash or recycled; they meet the definition of “hazardous waste.” Many consumers are unaware of this designation, but the EPA has provided guidance to separate batteries from other vape product components that are to be disposed of as municipal hazardous waste [23]. Furthermore, single-use or “disposable” vape products are not designed to be safely disassembled [24].

Disposable nicotine vape products are the most commonly sold [25] and used products in the United States [26], creating a growing environmental challenge [24]. In addition to fires caused by improper disposal of the lithium-ion batteries in vape products, the high-carbon impacts of vape product manufacturing are of concern [27]. The majority of young adults who use disposable nicotine vape products report throwing them in the trash when empty [24]. This provides an estimated 150 million disposable vapes (with potentially recoverable lithium) deposited annually in US landfills [27]. Nearly a quarter of young adults who use vape products report saving the empty products, which also presents a safety issue [24]. There have been no studies to our knowledge on disposal methods for cannabis vape products.

Cultivating public and political support for legislative and regulatory interventions for TECW requires increasing knowledge and modifying perceptions of TECW, and increasing public engagement with policies that can reduce use of tobacco and cannabis products [28]. Research is required on how to influence behaviors that support TECW regulatory policies. To date, there have been 2 observational studies on cigarette butt waste, which both showed that education promotes positive attitudes and behaviors regarding TECW regulations [28,29]. There has not yet been specific research conducted on interventions to increase knowledge, perceptions, and policy engagement on e-cigarette or cannabis product waste. A randomized controlled trial will provide important evidence on the efficacy of such interventions to address TECW, especially among young persons who generally are more engaged on environmental issues.

### Study Objectives

This study tests the administration of tobacco, e-cigarette, and cannabis waste education plus motivational and behavioral support (TECW Ed+) versus tobacco, e-cigarette, and cannabis waste education (TECW Ed) only on TECW among college-attending young adults (aged 18-25 years) to increase their engagement on TECW policy implementation. Brief education is standard of care in university settings, and given our finding that awareness of a technological resource is not sufficient for many people to use it [30], we sought to determine whether the addition of motivational enhancement and behavioral support to education would elicit action.

Outcomes include change in TECW knowledge, harm perceptions, attitudes toward regulation, and smoke- and vape-free regulatory policy engagement. Our overall goal is to identify an evidence-based educational intervention to address TECW, based on environmental concerns [31].

Such interventions are particularly relevant among young people, who are the critical stakeholders in planetary stewardship [32]. Students in California public universities are an important constituency for tobacco-related research given that the 23-institution California State University system and the 10-institution University of California system have 100% smoke- and vape-free campus policies, albeit with persistent policy violations [33]. Compliance issues at California public universities and colleges are emblematic of a broader smoke- and vape-free policy compliance problem at universities [34-36].

The specific study aims are to: first, assess knowledge and perceptions about TECW among participants randomized to the TECW Ed+ group compared to those randomized to the education-only control group (TECW Ed). It is hypothesized that at week 6, the TECW Ed+ group will demonstrate a significant increase in knowledge about TECW, perceived harmfulness of TECW, and the attitude that TEC manufacturers should be accountable for waste mitigation, and that the TECW Ed group will be noninferior to the TECW Ed+ group in these outcomes. Second, assess TECW-related behaviors among college students randomized to the TECW Ed+ group compared to those in the comparison group. It is hypothesized that at week 6, the TECW Ed+ group compared to the TECW Ed group will display greater usage of a campus-based TECW Tracker to report TECW on campus and stronger engagement in supporting smoke- and vape-free regulatory efforts. Third, document changes in campus TECW to understand the environmental impact of a TECW intervention. TECW on campus will be measured each week using 3 sources: Tracker-based participant reports, Tracker-based nonparticipant reports (eg, other members of the campus community), and repetitive objective environmental scans by the site team.

### Overview and Trial Design

This is a parallel randomized controlled trial at 2 California universities (1 University of California and 1 California State University) in which a digital TECW Tracker was previously deployed for reporting locations of smoking, vaping, and related waste on campus [30,37]. We enrolled 406 young adult students in a 6-week trial and randomly assigned them 1:1, stratified by site and tobacco or cannabis use status, to usual education only about TECW (TECW Ed) or to receiving the same education about TECW plus motivational enhancement and behavioral support for reporting TECW (TECW Ed+). Changes in individual-level and environmental outcomes are evaluated through repeated assessments at baseline, week 2, week 6, and week 26. Check-in text messages only are provided at weeks 1 and 4.

The guiding framework for this study is social cognitive theory [38,39]. in which behavior is conceptualized as the product of individual-level attitudes and perceptions and social structures that facilitate behavior change. In this study, we use motivational enhancement [40-47], goal setting [48-54], and problem-solving applications [55-60] as developed in our previous trial [61]. Our intervention was designed to address both individual-level attitudes and perceptions and social-level factors through the application of motivational enhancement, which is built around 4 main processes, carried out in a spirit of collaboration, respect, and support for the participant’s autonomy [54]. The first process, engaging, reflects fundamental communication principles and highlights the importance of empathy in establishing trust and rapport. The second process, focusing, involves working with participants to identify and agree on a topic for exploration. The third process, evoking motivation, is directed at drawing out and reinforcing participants’ own internal motivations for change. The fourth process, planning, guides participants in developing and implementing concrete steps toward their goals. The intervention protocol, in which motivational enhancement, goal setting, and problem solving were used to evoke changes in individual- and social-level factors per social cognitive theory, was adapted from one target behavior [61] to another (using TECW Tracker).

## Methods

### Ethical Considerations

The study was reviewed and approved by the Institutional Review Board at Site 1 (#1896205-1) on April 12, 2022. The study was reviewed and approved as exempt by the Institutional Review Board at Site 2 (#1952501-1) on September 2, 2022. Enrollment began on March 1, 2023, and concluded on October 15, 2024.

The informed consent form is delivered to participants by an emailed link in Research Electronic Data Capture (REDCap; Vanderbilt University), along with a link to schedule their baseline visit. The consent form asked, “Would you like to proceed with this study?” using a forced-choice question with two options: I do not wish to proceed with this study or I wish to proceed with this study. Written consent is provided through a typed name with acknowledgment of being aged 18 years or older, of having read and understood the study description, and that they may withdraw at any time. REDCap records the date of consent form completion.

At the beginning of the baseline visit, a description of the study, requirements, and compensation is reviewed; there is an opportunity to ask questions, and participants are asked whether they wish to proceed with the study. Any participants who have not completed the consent form prior to the visit are provided as much time as they need to read the form in a breakout room at the beginning of the visit.

**Figure 1:**
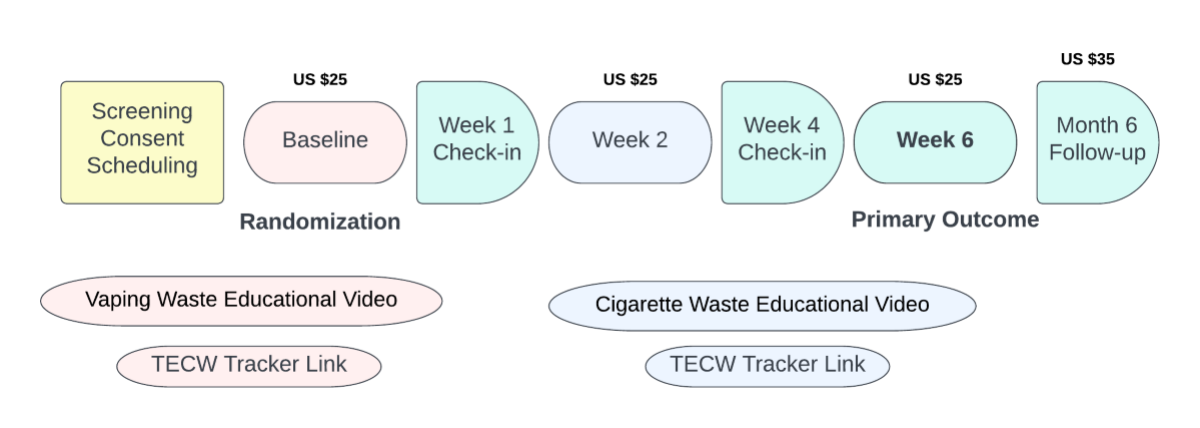
Visit flow.

Participants receive up to US $110 for participating in the study: US $25 for attending the baseline, week 2, and week 6 visits, and US $35 for the month 6 visit (Figure 1).

### Study Setting

The study is conducted in San Diego, California, United States (Site 1), and Davis, California, United States (Site 2). A project coordinator at Site 1 is responsible for participant recruitment and enrollment at both campuses and supervises Site 1–based research assistants. The intervention and assessments are delivered remotely by research assistants at Site 1 to participants at both sites. Participant education and TECW Ed+ group interaction occur over Zoom (Zoom Video Communications, Inc) as a one-to-one session with a research assistant. This method aligns with new norms for synchronous remote participation in university settings. Participants are provided links to surveys and forms in the Zoom chat and are placed in a breakout room to complete them. They are instructed to use the “ask for help” function or rejoin the main Zoom room with questions about the survey. The project coordinator reviews Tracker data and supervises staff at each site who conduct environmental scans and document TECW identified through the Tracker at both campuses.

### Eligibility Criteria

#### Inclusion Criteria

Study eligibility requirements include being a current student on the main campus of Site 1 or 2, attending class on the main campus at least once per week, having at least 6 months remaining until graduation, being aged between 18 and 25 years, English fluency, having a smart phone with location services enabled for apps, having regular access to a computer or tablet with sufficient Wi-Fi and a private space in which to complete remote study visits, and willingness to have the camera turned on during remote study visits.

#### Exclusion Criteria

The only exclusion criterion is prior study participation.

### Intervention Description

#### Overview

**Figure 2:**
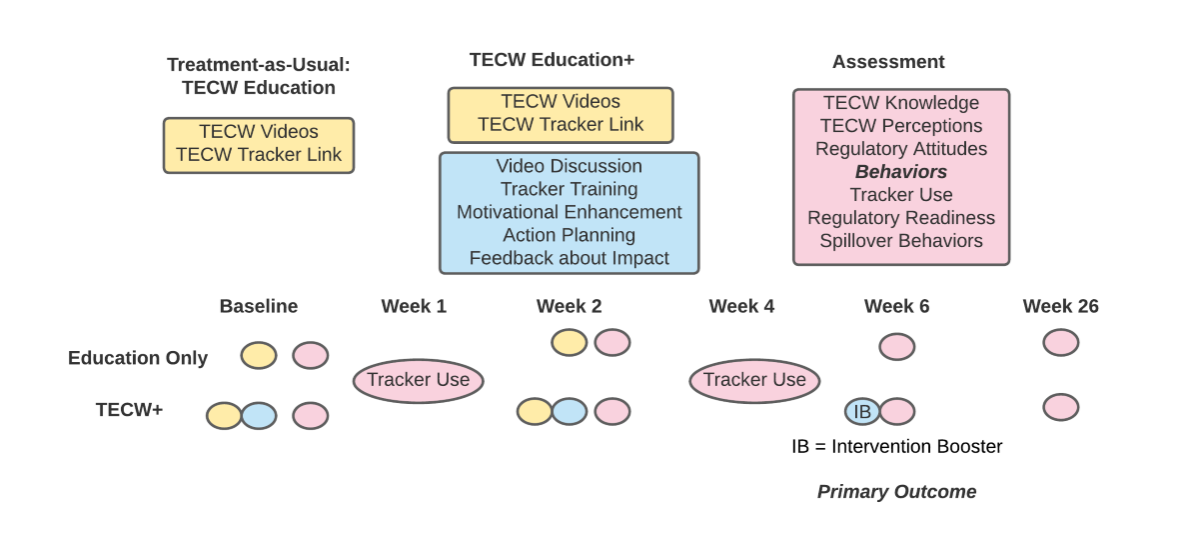
Intervention and assessment schedule.

Both groups receive education about TECW at baseline and week 2 visits by viewing videos and receiving a link to the TECW Tracker at baseline and week 2. The intervention group receives education plus motivational enhancement and behavioral support for using the TECW Tracker. Both groups receive a check-in text at week 1 and week 4 containing a brief assessment and a reminder for their next study visit. The intervention group’s text includes feedback about the positive environmental impact of making TECW reports and offers assistance with the Tracker if no reports had been made (Figure 2).

#### Education

Baseline education includes an overview of waste produced by vaping products used for tobacco and cannabis, their impact on the environment, and the role of tobacco and cannabis product manufacturers in TECW mitigation. This material is provided by a brief video from the California Youth Advocacy Network [62]. Week 2 education includes an overview of waste produced by cigarette smoking and the environmental impact of discarded butts. Tobacco Free CA provides brief videos [63,64]. The TECW Tracker is provided as a tool to identify TECW on the campus grounds; this then prompts TECW removal by research staff. The web link and QR code for the TECW Tracker app are provided, and participants are instructed to enter their study ID numbers when making a report.

The TECW Tracker is a digital tool that has been available since 2019 at Sites 1 and 2 to report smoking, vaping, and related waste on Site 1 and 2 campuses [30,37]. Tobacco Tracker provides three types of reports: (1) smoking- or vaping-related litter, (2) smoking or vaping, and (3) “looks good–no smoking or vaping or related litter.” Reports of litter from smoking or vaping allow identification of the source as cigarette butt, cigarette packaging, e-cigarette material (ie, pod, hardware), marijuana packaging, or other (select all that apply). Images of these litter types are included. Reports of smoking or vaping allow identification of the source as cigarette, e-cigarette or JUUL, marijuana (including blunts), do not know, or other; images of these products are embedded within the app. Reports of “looks good–no smoking or vaping or related litter” provide a way to monitor Tracker usage independent of problems and the capacity to measure improvement over time.

#### Mitigation of TECW

The project manager creates weekly mitigation assignments for site staff at each campus to address reports. Site staff conduct a 30-minute environmental scan of the location, collect TECW while wearing protective equipment, classify and document each piece of waste, and dispose of it properly. Each week, a randomly selected campus location is scanned for 60 minutes, and TECW is mitigated by site staff as described above.

#### Motivational Enhancement and Behavioral Support

Each study visit in which education is provided (baseline and week 2) contains additional motivational enhancement or behavioral support for the TECW Ed+ group. Each text message (week 1 and week 4) provides a brief assessment and reminder of the next study visit as well as additional motivational enhancement and behavioral support for the TECW Ed+ group. The week 6 visit provides another motivational booster to the TECW Ed+ group. Table 1 presents the components and timing of the motivational enhancement and behavioral support.

**Table 1:** Timing of motivational enhancement and behavioral support components.

Component	Baseline	Week 1	Week 2	Week 4	Week 6
Discuss video takeaways	✓		✓		
Discuss values and connect to Tracker	✓				
Instruction in using TECW^a^ Tracker	✓		As-needed^b^		
Offer assistance with using Tracker		As-needed^b^		As-needed^b^	
Motivation and confidence for using Tracker	✓				
Action planning for using Tracker	✓		✓		
Summary of Tracker reports and why reporting is useful		✓	✓	✓	✓

^a^TECW: tobacco, e-cigarette, and cannabis waste.

^b^Provided to those who have not made Tracker reports.

### Outcome Measurements

#### TECW Knowledge

Five items assess knowledge of nicotine and cannabis vape-related waste disposal methods (eg, Nicotine pods should be disposed as A. recyclable waste, B. hazardous waste [*correct], C. in normal trash, or D. in other ways) [65,66]. Six items assess knowledge of cigarette waste (eg, What is the main component of commercially sold cigarettes? A. cotton, B. paper, C. plastic [*correct], D. cork, or E. other [14]. Two general questions assessed biodegradability and recommended actions for dealing with TECW. A percent correct score is yielded for knowledge of vape-related and cigarette waste aligning with educational content at the baseline and week 2 visits, respectively.

#### TECW Perceptions

Six items assess agreement with various statements about cigarette and electronic devices used to vape nicotine and cannabis and their environmental impact including biodegradability; harm to the environment, animals, and sea life; and danger if thrown in trash [67,68], on a 5-point scale (strongly disagree to strongly agree). Participants are also asked how often they have noticed TECW in the past 30 days, which product type, and whether occurrences were on or off campus.

#### Regulatory Attitudes

Two items assess regulatory attitudes, including statements about whether various products (eg, cigarette filters, disposable products for vaping, pods or cartridges) should be included in bans of single-use plastic products. Response options range on a 5-point scale from strongly disagree to strongly agree. Tobacco manufacturer responsibility is assessed with items: How much responsibility do you think each of the following groups has for creating the problem of cigarette butt litter and should have for fixing the problem of cigarette butt litter? Groups include smokers, cigarette companies, city councils, and the government. Response options include none at all, a little, a moderate amount, and a great deal [67]. These same items are used to assess manufacturer responsibility for products used to vape nicotine and cannabis. While it may be impossible to distinguish whether vaping-related waste is from tobacco or cannabis, these items are assessed separately for tobacco and cannabis given different regulatory processes.

#### TECW Tracker Use

Awareness of a tool to report TECW on campus and use of the tool is assessed with yes or no questions [30]. Participant Tracker use is also measured objectively based on participant ID–linked TECW Tracker usage data.

#### Engagement in Smoke- and Vape-Free Regulatory Behavior

Engagement in smoke- and vape-free regulatory behavior is assessed with participant behaviors: (1) inform someone smoking or vaping on campus about the environmental impact of TECW, (2) inform someone smoking or vaping on campus about our smoke- and vape-free policy, (3) inform someone smoking or vaping on campus about how to properly dispose of TECW, (4) inform someone smoking or vaping on campus about a resource to quit smoking or vaping, (5) report TECW on campus, (6) report smoking or vaping on campus, and (7) volunteer (provide education, outreach, or clean-up) for campus smoke- and vape-free campus initiatives [30]. These items are rated from 1 (never expect to) to 5 (have already been doing) based on the transtheoretical model of behavior change [69].

#### Secondary Behaviors

To understand any broader impact of the intervention, related behaviors such as off-campus TECW advocacy are assessed; additionally, among tobacco users, proper disposal of e-waste and cessation are evaluated as secondary outcomes. The engagement in smoke- and vape-free regulatory behaviors is modified to “off campus” to assess this, and items about usual methods of TECW disposal and past 30-day quit attempts are assessed.

#### Environmental Impact of TECW Campus Intervention

Counts of TECW by product type as well as “looks good” reports are tabulated for each campus location on a weekly basis from the Esri ArcGIS–based TECW Tracker reports from participants and nonparticipants (eg, general campus community) through TECW Tracker. Counts of TECW by product type are also tabulated from objective environmental scans conducted by site staff.

As a secondary outcome, we assess the link between awareness of campus TECW mitigation and behavioral outcomes. Two items to assess this include: “How aware were you of how your Tracker reports were addressed?” (Response options include not at all, a little, moderately, and very aware), and “How satisfied were you with campus actions taken in addressing TECW?” (Response options range from very dissatisfied to very satisfied). An open-ended prompt follows for those who report dissatisfaction.

#### Attributions of Intervention Components

Following the week 6 survey, those in the TECW Ed+ group are administered another survey assessing their attributions of two intervention components: (1) talking with the study team and (2) receiving feedback about actions taken from TECW reports. They rate their agreement with the following three questions on a 5-point scale, ranging from strongly disagree to strongly agree: [Intervention Component]: (1) taught me new information about TECW, (2) increased my desire to do something to reduce TECW, and (3) increased my desire to report TECW.

### Assessment Schedule

Knowledge, perceptions, and regulatory attitudes for vape-related waste are tested before and after receiving education at the baseline visit. Knowledge, perceptions, and regulatory attitudes for cigarette waste are tested before and after receiving education at the week 2 visit. Knowledge, perceptions, and regulatory attitudes for vape-related waste and cigarette waste are retested at the week 6 and week 26 visits (Figure 2).

### Study Timeline

Enrollment is conducted from the second week of each semester or quarter until 6 weeks before final examinations week. Enrollment begins the second week of the semester to assure that past 7-day questions refer to a time of typical campus activity. When there is a campus break of 2 weekdays or longer, study visits are delayed by a week to adjust the past 7-day question time period for typical campus activity. The stop date each semester or quarter assures that the 6-week intervention is delivered while participants are still attending class on campus at least once per week.

### Sample Size

An initial sample size of 400 total (100 per group per campus) will detect a 15-16 percentage point difference in proportions with 80% power at the 0.05 level (2-sided), assuming a sample size of 380 at 6 weeks (95% retention) and 340 at 26 weeks (85% retention). This effect size is expected based on previous research on attitudes and behaviors [67]. This sample size also provides more than 95% power to detect noninferiority within a 0.5 SD margin at the 0.025 level (1-sided) or a difference in means of 0.5 SD at the 0.05 level (2-sided); 0.5 SD is considered a minimally important difference in health-related quality of life outcomes [70] and corresponds to 0.5 point difference on a 5-point scale with an SD of 1 point.

### Recruitment

Participants are recruited from each site using campus announcements, flyers, tabling, and word of mouth through campus partners.

### Assignment of Interventions: Allocation

After completing the baseline survey, participants at each site are randomized 1:1 to the two groups (TECW Ed or TECW Ed+) in blocks stratified by tobacco or cannabis user status (any use in the past 30 days: yes or no) by the project coordinator using REDCap, based on randomization tables created by the study statistician with computer-generated random numbers.

### Assignment of Interventions: Blinding

Research assistants are aware of group assignments and deliver the designated intervention. Participants are not told their group assignment. Research assistants are unaware of study hypotheses and do not see participants’ before and after survey responses. Research assistants are aware of the number of Tracker reports and their nature because providing feedback to the TECW Ed+ group is a component of the intervention.

### Data Collection and Management

At the baseline and week 2 visits, a presurvey is administered before education and a postsurvey is administered after education (plus motivational enhancement for the intervention group). Assessment windows are ±3 days for the week 1 check-in, ±1 week for the week 2 visit and week 4 check-in, ±4 weeks for the week 6 visit, and ±6 weeks for the week 26 visit.

#### Contact Attempts

We are using our proven methods for achieving over 80% retention up to 6 months, including multiple contact methods (ie, text, phone calls, emails), identifying multiple alternative contact people, and sending a digital greeting 2 months before the week 26 visit [71-74]. Up to 6 contact attempts, of alternating forms, are made. Contacting alternative contact people is the last effort to reach an unreachable participant.

#### Data Management

Participant data are collected and managed using REDCap tools that are hosted through Site 2. REDCap is a Health Insurance Portability and Accountability Act (HIPAA)–compliant, web-based application that supports data capture for research studies. All data collected are anonymous through assignment of participant ID numbers and password protected. REDCap data are stored securely on a server at Site 2. Environmental scan data are entered into Excel (Microsoft Corporation) and maintained on a shared drive.

### Statistical Methods

#### Primary Outcome

The primary outcome is change in knowledge, perceptions, and attitudes. The hypothesis that the TECW Ed+ group will demonstrate a significant increase in TECW knowledge, harm perceptions, and regulatory attitudes, and that the TECW Ed group will be noninferior to the TECW Ed+ group in these outcomes by a margin of half an SD, will be tested as follows. We will compute mean scores for change in knowledge, change in harm perception, and change in belief in each group along with 2-sided 95% CIs; a CI entirely above 0 indicates a statistically significant increase at the 5% level (2-sided). In addition, for each of these measures, we will compute the difference between the TECW Ed+ group and the TECW Ed group mean scores, along with a 1-sided 97.5% CI; a CI entirely below 0.5 SD indicates noninferiority of the TECW Ed group within a 0.5 SD margin at the 0.025 level (1-sided). We will also construct mixed-effects repeated measures models to assess knowledge, perceptions, and belief outcomes at weeks 2, 6, and 26 versus group, time, and a group × time interaction, controlling for site, tobacco or cannabis use status, demographics, and baseline level of the outcome variable.

#### Secondary Outcomes

The secondary outcomes are behavior changes. The hypothesis that the TECW Ed+ group, compared to the treatment-as-usual TECW Ed group, will display greater usage of the TECW Tracker and stronger engagement in supporting smoke- and vape-free regulatory efforts will be tested as follows. We will compare the TECW Ed+ group and the TECW Ed group with respect to the mean number of Tracker reports between baseline and 6 weeks using a generalized linear model with negative binomial distribution and log link. We will compare the 2 groups with respect to mean engagement score at 6 weeks using a 2-sample *t* test. We will also construct generalized linear mixed-effects repeated measures models to assess TECW-related behavior outcomes at weeks 1, 2, 4, 6, and 26 versus group, time, and a group × time interaction, controlling for site, tobacco or cannabis use status, demographics, and, for engagement, baseline level of the outcome variable. As an ancillary analysis, we will assess the link between awareness of campus TECW mitigation and behavioral outcomes among participants by including awareness as a covariate in models of Tracker report behavior. All randomized participants with week 6 data will be included in primary outcome analyses, and those who completed week 6 and month 6 will be included in follow-up analyses.

As an ancillary outcome, we will evaluate spillover behaviors such as off-campus TECW advocacy, and among tobacco users, proper disposal of e-waste and reported tobacco cessation by computing frequencies and percentages for each of these behaviors overall and by site and group at 6 weeks and 26 weeks. Furthermore, to describe changes in campus TECW, we will use generalized linear mixed models of the number of TECW reports per month to estimate trends over time and association with environmental scans, overall and among study participants, assuming autoregressive correlation from month to month at each site and negative binomial distribution with log link. Separate models will be created for different types of TECW, and interactions between site and time will be included to assess differences in trends between sites.

#### Missing Data

Although our follow-up rates in previous studies have typically exceeded 80%, missing data will arise for a variety of reasons (item nonresponse, etc). Prior to testing primary hypotheses, we will conduct exploratory analyses to determine if baseline characteristics predict patterns of missing data. Any significant (*P*<.10) predictors of missing data will be included as covariates in models above and used with multiple imputation methods [75,76] for evaluating model estimates.

### Oversight and Monitoring

#### Auditing

Data quality is managed in real time using daily checklists and verified for consistency in documentation across various sources.

#### Protections Against Risk

The study is classified as exempt by the institutional review board due to low risk from an educational intervention. Nonetheless, we safeguard participant privacy by ensuring they are in a private space during study visits, ensuring our study space is accessible only to human-subjects–certified study team members, and maintaining confidentiality of participant records through use of code numbers.

#### Debriefing

At the week 26 visit, participants are provided contact information to receive results of the study when complete and the website for the smoke- and vape-free initiative at each campus, and the opportunity to ask questions.

## Results

Data collection began in March 2023 and concluded in June 2025. Data analysis is expected to begin in late 2025, with final results anticipated for publication in summer 2026.

Results will be shared with academic and scientific audiences, and local, state, and national stakeholders through digital communications, community events, presentations, and publications. University audiences include academic units and administrative divisions at the participating institutions and broader university systems. Scientific outlets include peer-reviewed journal articles and conferences in areas of higher education and public health. Communications will be made with local, state, and national agencies and organizations conducting tobacco control work and environmental advocacy.

## Discussion

### Anticipated Principal Findings

The study uses a rigorous design to evaluate an educational intervention that can improve knowledge, attitudes, and behavior regarding tobacco and cannabis product waste. This improved knowledge and belief system is expected to support the implementation of tobacco- and vape-related environmental regulatory policies. Such interventions may increase smoke-free policy adherence and support efforts to enforce smoke- and vape-free campus policies. Educational efforts using an environmental lens, paired with a focus on industry accountability, may also support quitting efforts [77], contributing to long-term efforts to reduce the health and economic burdens of tobacco product use [78].

Universities are critical settings in which to prevent initiation of vaping, as a greater number of college students than noncollege students begin using e-cigarettes in young adulthood [79]. There are an estimated 19.9 million college students in the United States, providing an opportunity for a variety of targeted interventions to address the current epidemic of e-cigarette use. This investigation, involving a clinical trial of an intervention aiming to change what young adult college students know about TECW and how they can support policies to reduce this environmental problem, may stimulate similar research and policy implementation in other campus settings.

### Limitations

There are several limitations to note regarding this trial. First, while the study involved a single site in each public university system in California, these 2 sites are not necessarily representative of all public universities. Furthermore, state universities are not representative of other educational settings such as community colleges, private universities, and trade schools. Second, young college-attending adults aged 18-25 years were the target population for this study, assuming their more active interest in environmental protection [32]. However, the results may not generalize outside this age or educational group. Nevertheless, these young adults are more likely to initiate vaping then noncollege-attending young adults [79] and a need for improved smoke-free campus policy compliance in these settings was identified in previous research [34-36]. Finally, given that this is a very closely controlled trial of an educational intervention, it is uncertain if such an intervention will be effective in a real-time setting. The efficacy of the intervention is established in the controlled research setting, then followed by wider implementation and evaluation in the real world [31].

### Comparison With Prior Work

This study expands on previous studies addressing the harms of cigarette butt waste, to include nicotine- and cannabis-vape–related waste. This study also uses a more rigorous design than previous observational studies [28,29]. The common approach used by the previous observational studies and our randomized trial was education to increase knowledge about the environmental harm of tobacco product waste. We extended this approach by using brief videos to deliver additional messages on not only cigarette butt waste but also including vape products for nicotine and cannabis. This study is novel in testing whether motivational enhancement and behavioral support add value in creating behavior change versus using education only.

### Directions for Future Research

A follow up, larger implementation study would be a valuable next step to apply the intervention in various settings and conditions. Additional efficacy studies in different settings (eg, community college) and policy conditions (eg, colleges with designated smoking or vaping areas) would be beneficial. Our behavioral measures, such as Tracker usage and change in TECW disposal methods, will be central in planning future studies.

### Conclusions

This trial aims to evaluate educational tools to promote effective smoke- and vape-free campus policies. It investigates whether brief educational videos will increase knowledge of the environmental impact of TECW and change harm perceptions and attitudes toward regulatory policies on cigarettes and vape products. We will also learn whether education alone is sufficient to increase engagement in smoke- and vape-free regulatory policy support, or whether the addition of motivational enhancement and behavioral support is necessary to produce change. This study will be the first randomized controlled trial modifying cigarette waste perceptions and behaviors and is novel in its inclusion of nicotine- and cannabis-vape–related waste educational interventions.

## Abbreviations

HIPAA: Health Insurance Portability and Accountability Act
REDCap: Research Electronic Data Capture
TECW Ed: tobacco, e-cigarette, and cannabis waste education
TECW Ed+: tobacco, e-cigarette, and cannabis waste education plus motivational and behavioral support
TECW: tobacco, e-cigarette, and cannabis waste

## Data Availability

The datasets generated or analyzed during this study are available from the corresponding author on reasonable request.

## References

[1] Hendlin YH (2018). Alert: public health implications of electronic cigarette waste. Am J Public Health.

[2] Mock J, Hendlin YH (2019). Notes from the field: Environmental contamination from e-cigarette, cigarette, cigar, and cannabis products at 12 high schools – San Francisco Bay Area, 2018-2019. MMWR Morb Mortal Wkly Rep.

[3] (2021). Tobacco and the environment. Truth Initiative.

[4] (2021). A toxic, plastic problem: e-cigarette waste and the environment. Truth Initiative.

[5] Novotny TE (2019). Environmental accountability for tobacco product waste. Tob Control.

[6] Novotny TE, Lum K, Smith E, Wang V, Barnes R (2009). Cigarettes butts and the case for an environmental policy on hazardous cigarette waste. Int J Environ Res Public Health.

[7] Kabasci S (2020). Plastic waste and recycling. Biobased Plastics.

[8] Bonanomi G, Incerti G, Cesarano G, Gaglione SA, Lanzotti V (2015). Cigarette butt decomposition and associated chemical changes assessed by 13C CPMAS NMR. PLoS One.

[9] Novotny TE, Slaughter E (2014). Tobacco product waste: an environmental approach to reduce tobacco consumption. Curr Environ Health Rep.

[10] Slaughter E, Gersberg RM, Watanabe K, Rudolph J, Stransky C, Novotny TE (2011). Toxicity of cigarette butts, and their chemical components, to marine and freshwater fish. Tob Control.

[11] (2017). Tobacco and its environmental impact: an overview. World Health Organization.

[12] Landmark vote prohibits sale of filtered tobacco products [press release]. Santa Cruz County (CA).

[13] (2025). Intergovernmental negotiating committee on plastic pollution. United Nations Environment Programme.

[14] Patel M, Cuccia AF, Folger S, Benson AF, Vallone D, Novotny TE (2023). Support for cigarette filter waste policies among US adults. Tob Control.

[15] Chang H (2014). Research gaps related to the environmental impacts of electronic cigarettes. Tob Control.

[16] (2021). Plastic waste from tobacco and vaping products. Physicians for a Smoke-Free Canada.

[17] Leclerc SH, Badami MG (2022). Extended producer responsibility for e-waste management: policy drivers and challenges. Journal of Cleaner Production.

[18] Krause MJ, Townsend TG (2015). Hazardous waste status of discarded electronic cigarettes. Waste Manag.

[19] What a waste! Legally disposing of e-cigarettes and nicotine products. Public Health Law Center.

[20] Young KM Garbage from Washington state’s booming pot industry clogs gutters, sewers and landfills. The Washington Post.

[21] Smoke-free series: post-consumer waste of tobacco and vaping products. Public Health Ontario.

[22] Wartenberg AC, Holden PA, Bodwitch H, Parker-Shames P, Novotny T, Harmon TC, Hart SC, Beutel M, Gilmore M, Hoh E, Butsic V (2021). Cannabis and the environment: what science tells us and what we still need to know. Environ Sci Technol Lett.

[23] Tips for safe disposal of e-cigarettes and e-liquid waste. Environmental Protection Agency (EPA).

[24] Donovan EM, Azadi M, McKay T, Aarvig K, Kreslake J (2025). Not-so-disposable e-cigarettes: methods young people use to discard single-use e-cigarettes. Addiction.

[25] Monitoring U.S. e-cigarette sales: national trends data brief. CDC Foundation.

[26] Pulvers K, Jamalian N, Suh E, Faltaoos P, Stewart SL, Aston ER (2024). Nicotine and cannabis routes of administration and dual use among U.S. young adults who identify as Hispanic, non-Hispanic Black, and non-Hispanic White. Prev Med Rep.

[27] Chapman M, Johnston F Lithium being trashed by the tonne as disposable vapes flood the US market. The Bureau of Investigative Journalism.

[28] Hoek J, Gendall P, Blank M, Robertson L, Marsh L (2019). Butting out: an analysis of support for measures to address tobacco product waste. Tob Control.

[29] Turner J (2018). A component analysis of low-cost interventions to decrease cigarette butt litter in the natural environment. St Cloud State University, School of Health and Human Services.

[30] Pulvers K, Rice M, Stewart SL, Tong E (2022). Tobacco tracker: a new tool to support college smoke and tobacco free policies. Nicotine Tob Res.

[31] Kegler MC, Bundy L, Haardörfer R, Escoffery C, Berg C, Yembra D, Kreuter M, Hovell M, Williams R, Mullen PD, Ribisl K, Burnham D (2015). A minimal intervention to promote smoke-free homes among 2-1-1 callers: a randomized controlled trial. Am J Public Health.

[32] Pickering GJ, Schoen K, Botta M, Fazio X (2020). Exploration of youth knowledge and perceptions of individual-level climate mitigation action. Environ Res Lett.

[33] Chobdee J (2016). University of California, Center of Excellence for Smoke/Tobacco-Free Policy.

[34] Fallin-Bennett A, Roditis M, Glantz SA (2016). The carrot and the stick? Strategies to improve compliance with college campus tobacco policies. J Am Coll Health.

[35] Fallin A, Johnson AO, Riker C, Cohen E, Rayens MK, Hahn EJ (2013). An intervention to increase compliance with a tobacco-free university policy. Am J Health Promot.

[36] Baillie L, Callaghan D, Smith ML (2011). Canadian campus smoking policies: investigating the gap between intent and outcome from a student perspective. J Am Coll Health.

[37] Loureiro SF, Pulvers K, Gosdin MM, Clift K, Rice M, Tong EK (2021). The development of a web-based tobacco tracker tool to crowdsource campus environmental reports for smoke and tobacco-free college policies: mixed methods study. J Med Internet Res.

[38] Bandura A (2004). Health promotion by social cognitive means. Health Educ Behav.

[39] Islam KF, Awal A, Mazumder H, Munni UR, Majumder K, Afroz K, Tabassum MN, Hossain MM (2023). Social cognitive theory-based health promotion in primary care practice: A scoping review. Heliyon.

[40] Miller WR (2009). Motivational interviewing with problem drinkers. Behav Cogn Psychother.

[41] Miller WR, Rollnick S (2012). Motivational Interviewing: Helping People Change.

[42] Rollnick S, Miller WR (1995). What is motivational interviewing?. Behav Cogn Psychother.

[43] Steinberg ML, Ziedonis DM, Krejci JA, Brandon TH (2004). Motivational interviewing with personalized feedback: a brief intervention for motivating smokers with schizophrenia to seek treatment for tobacco dependence. J Consult Clin Psychol.

[44] Diclemente C, Velasquez M (2002). Motivational Interviewing and the Stages of Change.

[45] Resnicow K, McMaster F (2012). Motivational Interviewing: moving from why to how with autonomy support. Int J Behav Nutr Phys Act.

[46] Gersib JA, Rojo M, King SG, Doabler CT (2024). Motivational interviewing for students in school settings: a meta-analysis. J Sch Psychol.

[47] Fried TR, Yang M, Martino S, Iannone L, Zenoni M, Blakley L, O'Leary JR, Redding CA, Paiva AL (2022). Effect of computer-tailored print feedback, motivational interviewing, and motivational enhancement therapy on engagement in advance care planning: a randomized clinical trial. JAMA Intern Med.

[48] Locke EA, Latham GP (2002). Building a practically useful theory of goal setting and task motivation. A 35-year odyssey. Am Psychol.

[49] Hanley K, Zabar S, Charap J, Nicholson J, Disney L, Kalet A, Gillespie C (2014). Self-assessment and goal-setting is associated with an improvement in interviewing skills. Med Educ Online.

[50] Strecher VJ, Seijts GH, Kok GJ, Latham GP, Glasgow R, DeVellis B, Meertens RM, Bulger DW (1995). Goal setting as a strategy for health behavior change. Health Educ Q.

[51] Latham GP, Locke EA (1991). Self-regulation through goal setting. Organ Behav Hum Decis Process.

[52] Bodenheimer T, Handley MA (2009). Goal-setting for behavior change in primary care: an exploration and status report. Patient Educ Couns.

[53] Magill M, Martino S, Wampold BE (2022). Goal setting and monitoring with alcohol and other drug use disorders: principles and practices. J Subst Abuse Treat.

[54] Cole SA, Sannidhi D, Jadotte YT, Rozanski A (2023). Using motivational interviewing and brief action planning for adopting and maintaining positive health behaviors. Prog Cardiovasc Dis.

[55] Nezu AM, Maguth Nezu C, D'Zurilla TJ (2013). Problem-Solving Therapy: A Treatment Manual.

[56] Escoffery C, Mullen P, Genkin B, Bundy L, Owolabi S, Haardörfer R, Williams R, Savas L, Kegler M (2017). Coaching to create a smoke-free home in a brief secondhand smoke intervention. Health Educ Res.

[57] Sorsdahl K, Myers B, Ward CL, Matzopoulos R, Mtukushe B, Nicol A, Cuijpers P, Stein DJ (2014). Adapting a blended motivational interviewing and problem-solving intervention to address risky substance use amongst South Africans. Psychother Res.

[58] Lin H, Xu D, Yang M, Ma X, Yan N, Chen H, He S, Deng N (2022). Behaviour change techniques that constitute effective planning interventions to improve physical activity and diet behaviour for people with chronic conditions: a systematic review. BMJ Open.

[59] Nadal IP, Angkurawaranon C, Singh A, Choksomngam Y, Sadana V, Kock L, Wattanapisit A, Wiwatkunupakarn N, Kinra S (2024). Effectiveness of behaviour change techniques in lifestyle interventions for non-communicable diseases: an umbrella review. BMC Public Health.

[60] Lawlor ER, Islam N, Bates S, Griffin SJ, Hill AJ, Hughes CA, Sharp SJ, Ahern AL (2020). Third-wave cognitive behaviour therapies for weight management: a systematic review and network meta-analysis. Obes Rev.

[61] Pulvers K, Nollen NL, Rice M, Schmid CH, Qu K, Benowitz NL, Ahluwalia JS (2020). Effect of pod e-cigarettes vs cigarettes on carcinogen exposure among African American and Latinx smokers: a randomized clinical trial. JAMA Netw Open.

[62] The earth is not disposable: nicotine vape waste. California Youth Advocacy Network.

[63] Is tobacco bad for the environment?|Cigarette trash is a dirty problem (by an even dirtier industry). UNDO.

[64] Environmental impact of cigarette butts. American Nonsmokers' Rights Foundation.

[65] Russette HC, Harris KJ, Schuldberg D, Green L (2014). Policy compliance of smokers on a tobacco-free university campus. J Am Coll Health.

[66] Jancey J, Bowser N, Burns S, Crawford G, Portsmouth L, Smith J (2014). No smoking here: examining reasons for noncompliance with a smoke-free policy in a large university. Nicotine Tob Res.

[67] Hoek J, Gendall P, Blank M, Robertson L, Marsh L (2019). Butting out: an analysis of support for measures to address tobacco product waste. Tob Control.

[68] Epperson AE, Novotny TE, Halpern-Felsher B (2021). Perceptions about the impact of cigarette filters on the environment and smoking-related behaviors. J Adolesc Health.

[69] Prochaska JO, DiClemente CC, Norcross JC (1992). In search of how people change. Applications to addictive behaviors. Am Psychol.

[70] Norman GR, Sloan JA, Wyrwich KW (2003). Interpretation of changes in health-related quality of life: the remarkable universality of half a standard deviation. Med Care.

[71] Pierce JP, Strong DR, Stone MD, Villaseñor A, Dimofte CV, Leas EC, Oratowski J, Brighton E, Hurst S, Pulvers K, Kealey S, Chen R, Messer K (2020). Real-world exposure to graphic warning labels on cigarette packages in US smokers: the CASA randomized trial protocol. Contemp Clin Trials.

[72] Ahluwalia JS, Okuyemi K, Nollen N, Choi WS, Kaur H, Pulvers K, Mayo MS (2006). The effects of nicotine gum and counseling among African American light smokers: a 2 x 2 factorial design. Addiction.

[73] Nollen N, Befort C, Pulvers K, James AS, Kaur H, Mayo MS, Hou Q, Ahluwalia JS (2008). Demographic and psychosocial factors associated with increased fruit and vegetable consumption among smokers in public housing enrolled in a randomized trial. Health Psychol.

[74] Oren E, Pulvers K, Romero DR, Barber C, Carter E, Tracy LA, Novotny TE (2020). Effects of unfiltered cigarettes on smoking behavior and toxicant exposure: protocol for a randomized crossover clinical trial. JMIR Res Protoc.

[75] Barnard J, Meng XL (1999). Applications of multiple imputation in medical studies: from AIDS to NHANES. Stat Methods Med Res.

[76] White IR, Royston P, Wood AM (2010). Multiple imputation using chained equations: issues and guidance for practice. Stat Med.

[77] Ritchie D, Amos A, Martin C (2010). "But it just has that sort of feel about it, a leper"--stigma, smoke-free legislation and public health. Nicotine Tob Res.

[78] Chapman S (2006). Butt clean up campaigns: wolves in sheep's clothing?. Tob Control.

[79] Buu A, Hu Y, Wong S, Lin H (2020). Comparing American college and noncollege young adults on e-cigarette use patterns including polysubstance use and reasons for using e-cigarettes. J Am Coll Health.

